# Questionnaires on Perceptions of Artificial Intelligence in Health Care Among Health Care Students: Cross-Cultural Translation Into French and Linguistic Validation

**DOI:** 10.2196/76572

**Published:** 2026-03-19

**Authors:** Sylvain Kotzki, Calvin Massonnet Turner, Nicolas Vuillerme

**Affiliations:** 1 Grenoble INP, LIG Sangria, CNRS, UMR 5217, Université Grenoble Alpes, Bureau 315, Bâtiment Jean Roget, Campus Santé, Domaine de La Merci, Grenoble, 38706, France, +33 457422142; 2 Institut Universitaire de France, Paris, France

**Keywords:** health care students, artificial intelligence perception, AI perception, cross‑cultural adaptation, curriculum, linguistic validation, artificial intelligence, AI

## Abstract

**Background:**

Artificial intelligence (AI) is rapidly transforming health care by enhancing diagnostic accuracy, optimizing clinical workflows, and supporting decision-making across all health disciplines. As AI-driven tools are progressively introduced into health systems, educating future professionals about AI has become a critical priority to ensure safe, ethical, and effective use. Although several validated English-language questionnaires exist to assess medical students’ perceptions and readiness on AI in medicine, no French-language equivalents are currently available, which limits their use in francophone settings and hampers international comparisons. To bridge this gap and enable comparable, evidence-based assessment of AI perceptions among French health care students, rigorous cross-cultural adaptation of validated instruments is essential.

**Objective:**

This study aimed to translate, culturally adapt, and linguistically validate 5 established English-language questionnaires assessing medical students’ perceptions of AI in medicine to produce French versions suitable for subsequent psychometric validation and use across health care training programs.

**Methods:**

We followed international guidelines for the cross-cultural adaptation of self-report measures, combining independent forward translations, reconciliation, backward translation, expert committee review, and cognitive debriefing. Two bilingual translators first produced independent French versions of each questionnaire, which were reconciled into a single draft. A third bilingual translator, blinded to the original instruments, then performed backward translation into English. An expert panel reviewed all versions to ensure conceptual equivalence and to adapt items for applicability across health professions. Finally, cognitive testing was conducted with 38 French health care students (in medicine, pharmacy, adapted physical activity and health, nursing, and midwifery) to assess clarity, comprehensibility, and acceptability with iterative revisions made until consensus was reached.

**Results:**

During forward translation, wording discrepancies were observed for 73.6% (148/201) of expressions, but only 1.0% (2/201) of items required resolution due to meaning differences. In the backward translation step, 97.0% (195/201) of expressions were judged to be conceptually equivalent to the originals; the remaining 3.0% (6/201) of expressions were revised after discussion. Cognitive debriefing with students led to minor wording modifications in 26.4% (53/201) of expressions to improve clarity and readability without altering the underlying concepts.

**Conclusions:**

We produced French-language versions of 5 widely used questionnaires assessing health care students’ perceptions of AI in medicine, following a rigorous cross-cultural translation, adaptation, and linguistic validation process. These instruments preserve conceptual equivalence with their English originals and provide standardized tools to document AI-related knowledge, attitudes, and intentions among French-speaking health care students. This work lays the groundwork for subsequent psychometric studies of these French-language versions of questionnaires used in diverse health care training programs.

## Introduction

Artificial intelligence (AI) is revolutionizing health care by providing transformative tools for early detection, diagnosis, decision-making, therapeutic management, and patient follow-up [1-5]. Over the past 2 decades, the digitization of data and processes worldwide has propelled a massive transformation in patient care models and strategies [6]. However, integrating AI into health care implies more than just new technical tools; it demands a profound reorganization of health systems and clinical practices. While predictive medicine, which anticipates patient needs, and personalized medicine, which optimizes care from the moment of diagnosis, offer unprecedented prospects [7], this new landscape raises at least 2 crucial issues: trust in inherently opaque algorithms [8] and the responsibility of health professionals using them in health care [9].

Despite encouraging results from certain AI algorithms [10], numerous health care professionals remain cautious [11]. Indeed, over 60% report reluctance to adopt AI because of limited transparency [12], concerns regarding validation [13], and ethical [14] as well as security [15] risks. Asan et al [16] have shown that user training is key to fostering trust and clarifying responsibilities. Similarly, Yakar et al [17] demonstrate that practitioners with limited AI knowledge fear being replaced by automated systems, stressing the need for education that addresses both technical and ethical dimensions. Moreover, multiple studies highlight how specialized training programs can strengthen AI literacy, covering theoretical knowledge, practical skills, and ethical reflection, although the precise competencies to teach remain debated [18-20]. In France, such training programs are only beginning to be integrated into medical and paramedical curricula [21], revealing an initial lack of readiness that might hinder appropriate use and acceptance of AI.

Therefore, assessing health care students’ knowledge, perceptions, and motivations regarding AI is critical for designing targeted educational strategies. In other countries, surveys have already illuminated students’ expectations and concerns about AI in health care [22-24]. To achieve this goal, surveys based on validated questionnaires are necessary for gathering data that is both comparable and actionable. Thus far, several questionnaires have been specifically designed to assess medical students’ perceptions of AI in medicine. Among them, we selected five instruments as follows: (1) the Medical Artificial Intelligence Readiness Scale for Medical Students (MAIRS-MS) [25], (2) the questionnaire by Sit et al [26], (3) the questionnaire by Boillat et al [27], (4) the questionnaire by Li et al [28], and (5) the questionnaire by Park et al [29]. In their original form, these instruments provide robust conceptual frameworks for exploring AI readiness, perceived impact on specialties, behavioral intentions to learn about AI, and perceived barriers and risks, but they are all available only in English and have been developed in specific educational and cultural contexts.

First, the MAIRS-MS by Karaca et al [25] assesses readiness to integrate AI into clinical practice, focusing on theoretical knowledge, practical skills, and ethical considerations. Second, the questionnaire by Sit et al [26] explores UK medical students’ perceptions of AI, especially its impact on radiology, by probing both their optimism and concerns regarding technological substitution. Third, Boillat et al [27] offer a questionnaire designed to identify both obstacles and facilitators to AI adoption among health care professionals and medical students, addressing challenges such as algorithmic complexity and the risk of dehumanizing care. Fourth, Li et al [28] investigate factors influencing medical students’ intentions to pursue AI training, taking into account social pressure, the perceived usefulness of AI in medicine, and self-confidence in mastering the technology. Finally, Park et al [29] delve into how AI shapes medical students’ perceptions of medical specialties and their career choices by assessing their confidence levels and primary sources of information about these emerging technologies.

Collectively, these 5 questionnaires [25-29], which are specifically addressed to medical students, provide a comprehensive and complementary perspective on the challenges and opportunities related to training future health care professionals in AI. However, since they are currently available only in English, their application to French-speaking settings remains limited. Moreover, none of these instruments has yet undergone a systematic cross-cultural linguistic adaptation for French-speaking health care students, which prevents their direct use for curriculum development and comparative research in francophone contexts.

In this context, our study aimed to translate, culturally adapt, and linguistically validate 5 key instruments, producing French versions that are conceptually equivalent to the originals and suitable for use across different health training programs. In addition to these conceptual and educational challenges, the rigorous adaptation of perception questionnaires requires a structured cross-cultural procedure (translation, cultural adaptation, and linguistic validation) distinct from subsequent psychometric validation, as recommended in international guidelines for self-report measures [30]. Although the original instruments were designed for medical students, we adapted the wording so that they can be administered to students from different health professions in the French context; however, their psychometric properties and applicability across diverse health training programs will need to be confirmed in subsequent studies.

## Methods

The transcultural adaptation of the questionnaires into French was carried out following international guidelines for the cross-cultural adaptation of self-report measures, combining independent forward translations, backward translation, expert committee review, and cognitive debriefing [30-32]. Operationally, the procedure was implemented using a methodology adapted from Epstein et al [33], previously applied to French-language instruments.

### Ethical Considerations

This study has received ethics approval from the Ethics Committee for the Integrity and Ethics of Research in Health Professions Education of the Société Internationale Francophone d’Education Médicale (1210-2023, issued on November 26, 2023).

This study corresponded to a qualitative cognitive debriefing or pilot linguistic validation phase (ie, the validation of version D of each questionnaire, the version sent to students), aimed at assessing the clarity, comprehensibility, and acceptability of translated items. In accordance with applicable French regulations for minimal-risk educational research, written informed consent was not required. Participants were required to read the study information sheet before participation, and oral informed consent was obtained prior to collecting their feedback. Participants could decline or withdraw at any time without consequences for their training or assessment.

Feedback was collected and analyzed for the sole purpose of improving linguistic clarity and conceptual equivalence of the French versions. Data were handled confidentially, with access restricted to the research team. The study followed institutional procedures and applicable French data protection requirements (law No. 78-17 of 6 January 1978, as amended), including provisions related to the Commission nationale de l'informatique et des libertés, where applicable. Results are reported in the aggregate, and no identifying information is disclosed in this paper.

No compensation, payment, or academic credit was provided.

### Questionnaires

Five questionnaires were targeted for cross-cultural validation in this study. The first questionnaire, MAIRS-MS, proposed by Karaca et al [25], evaluates the readiness of Turkish medical students to integrate AI into their future clinical practice. It includes 22 items distributed among 4 factors: cognitive (8 items), aptitude (8 items), vision (3 items), and ethical (3 items). Responses are recorded on a 5-point Likert scale ranging from 1 (“strongly disagree”) to 5 (“strongly agree”), yielding a total score between 22 and 110 points, with higher scores indicating greater readiness for AI integration in medical practice.

The second questionnaire, developed by Sit et al [26], explores UK medical students’ perceptions of AI, with particular emphasis on its impact on radiology. It includes 15 questions in total, 11 of which are formatted as a 5-point Likert scale ranging from 1 (“strongly disagree”) to 5 (“strongly agree”) and 1 with a 5-point Likert scale ranging from 1 (“extremely useful”) to 5 (“not at all useful”). These items assess medical students’ knowledge of AI, their perceptions of its role in the evolution of the medical profession, and their potential apprehensions about technological substitution.

The third questionnaire, proposed by Boillat et al [27], consists of 17 questions designed to measure the familiarity of medical students and physicians with AI and to identify perceived barriers to its adoption in clinical practice. This questionnaire is structured around four themes: (1) familiarity with AI; (2) AI training; (3) factors facilitating AI implementation, including obstacles such as the complexity of algorithms, data availability, and trust in AI tools; and (4) risks associated with AI development, such as the dehumanization of care or the gradual loss of clinical skills. Three of the themes are formatted as a 5-point Likert scale ranging from 1 (“strongly disagree”) to 5 (“strongly agree”), and 1 theme is formatted as a 5-point Likert scale ranging from 1 (“never heard of it”) to 5 (“can confidently explain it”).

The fourth questionnaire, proposed by Li et al [28], comprises 23 items that examine the factors influencing the motivation of medical students to learn about AI and integrate these technologies into their training. This questionnaire includes 5 subscales: the perceived relevance of AI in medicine, the influence of peers and teachers, confidence in one’s ability to understand and use AI, basic knowledge of AI, and behavioral intention to learn AI. Responses are recorded on a 6-point Likert scale ranging from 1 (“not true of me at all”) to 6 (“very much true of me).

Finally, the fifth questionnaire, proposed by Park et al [29], focuses on medical students’ perceptions of the impact of AI on medical practice and its influence on specialty choice. It seeks to assess the extent to which AI affects students’ career prospects and includes 6 multiple-choice questions, 2 Likert-scale items, and open-ended questions to capture qualitative insights.

### Forward Translation

The forward translation of each questionnaire from the original English into French was performed by 2 independent bilingual translators. One translator (translator 1) was a native English speaker, while the other (translator 2) was a native French speaker. Translator 1 was familiar with the concepts evaluated by the 5 questionnaires, while translator 2 was not informed of their intended purpose. The 2 translations were later compiled, and in cases of disagreement, the 2 translators were consulted to reach an agreement. Each questionnaire was then thoroughly reviewed to correct errors in spelling, grammar, and punctuation and to ensure that terminology and style were faithful to the original English version.

### Backward Translation

Following this, backward translation was conducted. Version A of each questionnaire was translated from French back into English (version B) by a bilingual translator whose native language was English (back translator) and who had no expertise in health care or AI. The back translator was instructed to translate each questionnaire literally. The original English version and the back-translated version were then compared and reconciled to produce a synthesized French version (version C), ensuring translation accuracy and highlighting any potentially confusing wording.

### Adaptation of Questionnaires to All Health Training Courses

Version C was then submitted to a health professional to propose adjustments that would extend the applicability of the questionnaires to all health training courses. For each questionnaire, this health professional was asked to suggest modifications when questions were considered exclusively proper for physician training or medical practice, thereby producing an extended version (version D).

### Final Validation

The final validation phase involved conducting a preliminary pilot test with native French-speaking participants representative of the target population to assess the clarity, intelligibility, and acceptability of the translations. Between October 10, 2024, and December 1, 2025, version D was sent to 38 students enrolled in various initial health training programs in France (women: n=27, 71.1%; men: n=11, 28.9%), including pharmacy (n=10, 26.3%), medicine (n=8, 21.1%), adapted physical activity and health (n=8, 21.1%), nursing (n=6, 15.8%), and midwifery (n=6, 15.8%) programs. They were asked to review the intelligibility of the questions and the proposed answers in version D, and their feedback was synthesized to produce version E.

### Data Analysis

All qualitative feedback obtained during the final verification phase was analyzed using a thematic approach. Comments were independently coded by 2 researchers to identify issues related to clarity, comprehension, terminology, and formatting. Any discrepancies in coding were resolved through discussion until consensus was reached. On the basis of these findings, problematic items were iteratively revised to enhance semantic equivalence with the original questionnaires and improve readability in French. No quantitative statistical analyses were conducted during this phase, as the primary objective was to ensure content validity and linguistic clarity of the translated questionnaires.

## Results

### Characteristics of the Translators and the Participants

The age, gender, and specific cultural characteristics of the translators and the student participants are detailed in Table 1.

**Table 1.** Age, gender, and cultural characteristics of the translators and the participants.

Step	Number of translators or participants	Age (y), mean (SD; range)	Gender	Cultural characteristics
Forward translation	2 translators	41.5 (13.5; 28-55)	1 man and 1 woman	1 native English speaker and 1 native French speaker; 1 familiar with the concept of AI^a^ and 1 unfamiliar with the concept of AI
Backward translation	1 translator	37 (—^b^)	1 woman	1 native English speaker
Adaptation of health studies	1 expert	40 (—)	1 man	1 health care professional with more than 10 years of experience
Final verification	38 students	23.6 (5.57; 18-50)	27 women and 11 men	10 pharmacy students, 8 medical students, 8 adapted physical activity and health care students, 6 nursing students, and 6 midwifery students

a AI: artificial intelligence.

b Not applicable.

### Forward Translation

Across the 5 instruments, the 2 forward translators proposed identical wording for 26.4% (53/201) of the expressions, whereas 73.6% (148/201) of the expressions differed in vocabulary, register, or syntax. In almost all cases, these discrepancies did not affect the underlying meaning, and a consensual French wording was obtained through discussion and recontextualization of the items. Only 2 items required arbitration because the alternative translations conveyed slightly different concepts. For example, for the MAIRS-MS item “I find valuable to use AI for education,” translator 1 suggested “J’estime qu’il est utile d’utiliser l’IA à des fins d’éducation,” whereas translator 2 proposed “Je trouve valorisant l’utilisation de l’IA à des fins de pédagogie.” After discussion, the first option was retained because it emphasized the perceived usefulness of AI in education rather than the personal gratification implied by “valorisant.” Similarly, for 1 item in the questionnaire by Sit et al [26] concerning students’ anticipated understanding of methods to evaluate AI algorithm performance, the agreed wording (“je maîtriserai davantage les méthodes utilisées pour évaluer les performances des algorithmes d’IA dans la santé”) was preferred over alternatives focusing on “connaissance” alone to reflect the level of competence implied in the original English version. In the remaining items, minor stylistic variations (eg, “Je peux...” vs “Je suis capable de...”) were resolved in favor of the simplest and most natural phrasing for health care students, without altering the constructs being measured.

### Backward Translation

When the back-translated English versions (version B) were compared with the original questionnaires, no loss or change in meaning was identified for 3 instruments (Karaca et al [25], Sit et al [26], and Park et al [29]). Overall, 97.0% (195/201) of the expressions were judged to be conceptually equivalent to the originals, and only 3.0% (6/201) of the expressions required revision after discussion with the back translator. In the questionnaire by Boillat et al [27], only 1 expression exhibited a variation in meaning between translator 3’s version and the original version. The proposals “Do you think it is necessary to receive more in-depth training in order to” from version B and “Would you benefit from more training to” from the original version were discussed with the back translator, which resulted in replacing the expression “Pourriez-vous bénéficier d’une formation plus poussée pour” (“Could you benefit from further training for”) in version A with “Pensez-vous nécessaire de bénéficier d’une formation plus approfondie pour” (“Do you think it is necessary to benefit from more in-depth training to”) in version C. In the questionnaire by Li et al [28], 5 phrases showed meaning discrepancies and led to minor wording adjustments in the French version to better reflect the original notions of “health care solutions,” future concerns about the development of medical AI, and actual interaction with medical AI applications (eg, replacing “les solutions de soins” [“care solutions”] with “les solutions de santé” [“health solutions”] and “J’ai étudié des applications médicales” [“I have studied medical apps”] with “J’ai interagi avec des applications médicales” [“I have used medical apps”]). All remaining differences between the back-translated and original versions were considered stylistic and did not require changes to the French wording.

### Adaptation of Questionnaires to All Health Training Courses

The health professional who reviewed version C proposed several modifications to extend the scope of the 5 questionnaires to all students in health training. For all questionnaires, and when no change in meaning or interpretation was involved, the health professional systematically proposed the following substitutions: “médecine” (“medicine”) was replaced with “santé” (“health”), “médecins” (“physicians”) was replaced with “professionnels de santé” (“health professionals”), “radiologie” (“radiology”) was replaced with “imagerie médicale” (“medical imaging”), “diplôme de médecine” (“medical degree”) was replaced with “diplôme de professionnel de santé” (“health professional degree”), “pratiques cliniques” (“clinical practices”) was replaced with “pratiques professionnelles” (“professional practices”), and “applications médicales” (“medical applications”) was replaced with “applications en santé” (“applications in health”). In the questionnaire by Boillat et al [27], the list of medical specializations was replaced by a list of “professions de santé” (“health professions”) to align with the objective of creating an extended version of the questionnaire. All these modifications were later incorporated into version D of each questionnaire.

### Final Validation

The extended versions of the 5 questionnaires (version D) were submitted to 38 native French-speaking health care students from 5 different health training programs (pharmacy, medicine, adapted physical activity and health, nursing, and midwifery). Each student was invited to propose modifications to ensure the intelligibility of both the questions and the response options. A total of 53 expressions were subject to revision proposals for improved clarity. For example, the phrase “Je peux établir des plans de travail compatibles avec l’IA” (“I can build AI-enabled worktops”) was replaced with “Je suis capable d’organiser des flux de travail compatibles avec l’intelligence artificielle” (“I’m able to organize AI-enabled workflows”), a version deemed more intelligible by the student panel. The complete French versions of the 5 questionnaires are available in Multimedia Appendix 1.

## Discussion

### Principal Findings

In this study, we translated, culturally adapted, and linguistically validated 5 established English-language questionnaires [25-29] to assess French health care students’ perceptions of AI, using a rigorous forward-backward translation process, expert arbitration, and cognitive testing with 38 French students from 5 different health training programs (pharmacy, medicine, adapted physical activity and health, nursing, and midwifery). We produced French versions that maintain strong conceptual equivalence with the original versions while ensuring clarity and accessibility for an academic audience.

The health care sector is undergoing a profound digital transformation, making AI literacy an essential competency for future health professionals [18,19]. Capturing the in-depth views, knowledge, and expectations of French health care students on AI is a crucial first step, and perception surveys provide an effective means to gather these insights. For instance, a 2020 European survey of 451 medical students found that although 40% were preparing for an increasingly digitized health system, more than half felt inadequately trained in digital technologies and nearly 85% called for enhanced digital health education in the medical curriculum [22]. Similarly, in the United States, a study published in 2022 revealed that 91.5% of the 390 surveyed medical students believed that AI training would be beneficial for their future practice, yet 91.2% reported that their institutions provided little or no formal instruction on the subject [24]. A 2022 Canadian survey involving 2167 students across 10 health disciplines indicated that 79% of respondents anticipated that AI would impact their careers within the next decade [23]. Even initially skeptical students recognize the need to acquire fundamental AI skills in their training, despite some concerns, for example, an Austrian study highlighted skepticism among medical students who viewed digitalization as a potential threat to the physician-patient relationship [34]. Collectively, these studies highlight both a strong demand for AI education and persistent concerns regarding its impact on professional roles.

Insights from these surveys can directly inform curriculum design by prioritizing fundamental AI concepts, ethical and legal issues, and practical hands-on training. European medical students expressed a desire for dedicated courses addressing health data management, ethical and legal issues, and other digital health topics, alongside more practical, hands-on training through workshops or exercises that illustrate the use of AI and digital health tools [22]. Similarly, American medical students favored learning formats such as short lectures (70%), optional dedicated courses (48%), and question and answer sessions with experts (44%), and they emphasized the need to focus on fundamental AI concepts and criteria for its application in medicine rather than solely on technical aspects [24].

In this context, our French adaptations of 5 complementary questionnaires [25-29] fill an important gap in French-speaking contexts by providing, for the first time, a coherent set of standardized tools that, once fully validated, will support the systematic assessment of AI readiness, perceived impact on specialties, behavioral intentions to engage with AI, and perceived barriers and risks among health care students. Beyond enabling cross-sectional surveys, these instruments can also be used longitudinally to monitor changes in perceptions over time, compare cohorts or curricula, and evaluate the impact of new educational interventions related to AI and digital health.

### Limitations

This study has several limitations that must be acknowledged. First, the cognitive testing was conducted with a convenience sample of 38 students from 5 different health training programs within a single institution. Although this multiprofessional sample improves the face validity and perceived applicability of the French versions across health curricula, it remains a single-center, relatively small pretest, typical of exploratory cognitive debriefing in cross-cultural adaptation studies and comparable to previous work using similar forward-backward methodologies [35,36]. Second, we did not perform any psychometric analyses (eg, internal consistency, factor structure, and construct validity) in this study; these will be the focus of subsequent validation work.

### Future Research

Future work should build on this linguistic validation by conducting full psychometric evaluations of the French versions of these instruments in larger and more diverse samples of health care students. Key steps will include assessing internal consistency and test-retest reliability; examining the factor structure of each questionnaire through exploratory and confirmatory factor analyses; and investigating construct validity by relating scores to relevant constructs such as digital health literacy, previous exposure to AI, or attitudes toward technology. Future studies should follow established methodological standards for evaluating the measurement properties of self-report instruments, such as those proposed in the Consensus-Based Standards for the Selection of Health Measurement Instruments and in classical psychometric frameworks for scale development [37]. Multiprofessional and multicenter studies will also be essential to examine the generalizability of these instruments across different health professions and educational settings, thereby extending the preliminary multiprofessional cognitive testing conducted in this study.

Beyond cross-sectional validation, future studies could also explore measurement invariance across disciplines and institutions and assess the responsiveness of these instruments to educational interventions introducing AI and digital health content into curricula. In the longer term, empirical data from these validation studies may support the development of a shortened, transdisciplinary questionnaire that captures core AI-related perceptions and readiness across health professions, suitable for routine use in curriculum design and longitudinal monitoring of AI literacy in health education.

### Conclusions

In this study, we translated, culturally adapted, and linguistically validated into French 5 established English-language questionnaires originally designed to assess medical students’ perceptions of AI in health care, and we adapted their wording for use among health care students in the French context. These adaptations fill an important gap in francophone contexts by providing standardized, conceptually equivalent instruments to document AI-related knowledge, attitudes, and intentions across health training programs. Once fully validated through psychometric studies in larger and more diverse samples, these questionnaires could be used in a robust manner for curriculum design, evaluation of educational interventions, and international comparisons of AI literacy in health education.

## Supplementary material

10.2196/76572 Multimedia Appendix 1 Translation and linguistic validation in French of the 5 questionnaires.

## Abbreviations

AI: artificial intelligence
MAIRS-MS: Medical Artificial Intelligence Readiness Scale for Medical Students
