# Supplementary material for: Questionnaires on Perceptions of Artificial Intelligence in Health Care Among Health Care Students: Cross-Cultural Translation Into French and Linguistic Validation
Source: JMIR Med Educ. 2026 Mar 19;12:e76572. doi: 10.2196/76572 (PMC13002006; doi:10.2196/76572)
Supplement: Multimedia Appendix 1 [file mededu-v12-e76572-s001.docx]

## Multimedia Appendix

Table S1. Translation and linguistic validation in French of the questionnaire from Karaca

| MEDICAL ARTIFICIAL INTELLIGENCE READINESS SCALE FOR MEDICAL STUDENTS (MAIRS‑MS) | |
| --- | --- |
| The original English version | The final version translated into French |
| 1-Strongly Disagree  2-Disagree  3-Neutral  4-Agree  5-Strongly Agree | 1-Pas du tout d’accord  2-Pas d’accord  3-Sans opinion  4-D’accord  5-Tout à fait d’accord |
| I can define the basic concepts of data science | Je peux définir les concepts de base de la science des données. |
| I can define the basic concepts of statistics | Je peux définir les concepts de base en statistiques. |
| I can explain how AI systems are trained | Je peux expliquer comment les systèmes d’intelligence artificielle sont entraînés. |
| I can define the basic concepts and terminology of AI | Je peux définir les concepts et les terminologies de base en intelligence artificielle. |
| I can properly analyze the data obtained by AI in healthcare. | Je peux analyser correctement les données obtenues par l'intelligence artificielle dans le domaine de la santé. |
| I can differentiate the functions and features of AI related tools and applications. | Je peux différencier les fonctions et les caractéristiques des outils et des applications liés à l'intelligence artificielle. |
| I can organize workflows compatible with AI. | Je suis capable d'organiser des flux de travail compatibles avec l'intelligence artificielle |
| I can express the importance of data collection, analysis, evaluation and safety; for the development of AI in healthcare. | Je peux exprimer l'importance de la collecte, de l'analyse, de l'évaluation et de la sécurité des données pour le développement de l'intelligence artificielle dans la santé. |
| I can harness AI-based information combined with my professional knowledge. | Je peux exploiter des informations issues de l’intelligence artificielle en les combinant avec mes connaissances professionnelles. |
| I can use AI technologies effectively and efficiently in healthcare delivery. | Je peux utiliser les technologies de l'intelligence artificielle de manière efficace et efficiente dans la prestation de soins. |
| I can use artificial intelligence applications in accordance with its purpose. | Je peux utiliser les logiciels d'intelligence artificielle conformément à leur objectif. |
| I can access, evaluate, use, share and create new knowledge using information and communication technologies. | Je peux accéder, évaluer, utiliser, partager et créer de nouvelles connaissances en utilisant des technologies d’information et de communication. |
| I can explain how AI applications offer a solution to which problem in healthcare. | Je peux expliquer comment les logiciels d'intelligence artificielle offrent une solution adaptée à un problème de santé précis. |
| I find valuable to use AI for education, service and research purposes. | J'estime qu'il est utile d'utiliser l'intelligence artificielle à des fins d'éducation, de service et de recherche. |
| I can explain the AI applications used in healthcare services to the patient. | Je peux expliquer aux patients les logiciels d'intelligence artificielle utilisées dans les services de soin. |
| I can choose proper AI application for the problem encountered in healthcare. | Je peux choisir les logiciels d'intelligence artificielle correspondant aux problèmes rencontrés dans le domaine de la santé. |
| I can explain the limitations of AI technology. | Je peux expliquer les limites des technologies de l'intelligence artificielle. |
| I can explain the strengths and weaknesses of AI technology. | Je peux expliquer les forces et les faiblesses des technologies de l’intelligence artificielle. |
| I can foresee the opportunities and threats that AI technology can create. | Je peux envisager les opportunités et les menaces que les technologies de l’intelligence artificielle peuvent engendrer. |
| I can use health data in accordance with legal and ethical norms. | Je peux utiliser les données relatives à la santé dans le respect des normes juridiques et éthiques. |
| I can conduct under ethical principles while using AI technologies. | Je peux suivre les principes éthiques lorsque j'utilise l’intelligence artificielle. |
| I can follow legal regulations regarding the use of AI technologies in healthcare. | Je peux suivre les réglementations légales concernant l'utilisation de l'intelligence artificielle dans le domaine de la santé. |

Table S2. Translation and linguistic validation in French of the questionnaire from Sit.

| QUESTIONNAIRE ON CURRENT ATTITUDES AND UNDERSTANDING ON ARTIFICIAL INTELLIGENCE OF MEDICAL STUDENTS IN THE UNITED KINGDOM | |
| --- | --- |
| The original English version | The final version translated into French |
| 1-Strongly Agree 2-Agree  3-Neutral  4-Disagree  5-Strongly Disagree | 1-Tout à fait d'accord 2-D’accord  3-Sans opinion  4-Pas d’accord  5-Pas du tout d’accord |
| Which Medical School do you currently attend? | Dans quelle faculté ou école de santé étudiez-vous actuellement ? |
| Please rate your agreement to the follow questions: | Veuillez indiquer si vous êtes d'accord avec les affirmations suivantes : |
| AI will play an important role in healthcare | L'intelligence artificielle jouera un rôle important dans le domaine de la santé |
| I am LESS likely to consider a career in radiology, given the advancement of AI | Je suis MOINS enclin à envisager une carrière en radiologie, compte tenu des progrès de l'intelligence artificielle. |
| Some specialties will be replaced by AI during my lifetime | Certaines spécialités seront remplacées par l'intelligence artificielle au cours de ma vie. |
| I have an understanding of the basic computational principles of AI | Je comprends les principes informatiques de base de l'intelligence artificielle. |
| I am comfortable with the nomenclature related to artificial intelligence | Je suis à l'aise avec la nomenclature relative à l'intelligence artificielle. |
| I have an understanding of the limitations of artificial intelligence | Je comprends les limites de l'intelligence artificielle. |
| Teaching in artificial intelligence will be beneficial for my career | L'enseignement de l'intelligence artificielle sera bénéfique pour ma carrière de professionnel de santé. |
| All medical students should receive teaching in artificial intelligence | Tous les étudiants en faculté ou en école de santé devraient recevoir un enseignement sur l'intelligence artificielle. |
| At the end of my medical degree, I will be confident in using basic healthcare AI tools if required | À la fin de mon cursus en école de santé, je serai capable d'utiliser en confiance des outils d'intelligence artificielle dans le domaine de la santé, si nécessaire. |
| At the end of my medical degree, I will have a better understanding of the methods used to assess healthcare AI algorithm performance | À la fin de mon cursus en santé, je comprendrai mieux les méthodes d'évaluation des performances des algorithmes d'intelligence artificielle en santé. |
| Overall, at the end of my medical degree, I feel I will possess the knowledge needed to work with AI in routine clinical practice | A la fin de mon cursus en école de santé, je pense que j’aurai les connaissances nécessaires pour travailler avec l'intelligence artificielle dans la pratique clinique courante. |
| Please answer yes or no to the following questions: | Veuillez répondre par oui ou par non aux questions suivantes : |
| I have received teaching/training in artificial intelligence | J'ai reçu un enseignement ou une formation en intelligence artificielle |
| o Yes o No | Oui - Non |
| If you have answered yes to the previous question, was this teaching/training a compulsory part of your medical degree? | Est-ce-que cet enseignement ou cette formation était obligatoire dans votre cursus en école de santé ? |
| o Yes o No o N/A | Oui - Non - Je ne sais pas |
| If you have received teaching/training in artificial intelligence, please rate the usefulness of the teaching/training you have received | Veuillez évaluer l'utilité de cet enseignement ou de cette formation. |
| o Extremely useful o Very useful o Somewhat useful o Not so useful o Not at all useful | o Extrêmement utile o Très utile o Assez utile o Peu utile o Pas du tout utile |

Table S3. Translation and linguistic validation in French of the questionnaire from Boillat

| READINESS TO EMBRACE ARTIFICIAL INTELLIGENCE AMONG MEDICAL DOCTORS AND STUDENTS | |
| --- | --- |
| The original English version | The final version translated into French |
| What is your gender? | Quel est votre genre ? |
| Female | Féminin |
| Male | Masculin |
| Prefer not to say | Préfère ne pas répondre |
| What is your age group? | Quelle est votre tranche d’âge ? |
| <20 | < 20 ans |
| 20-29 | 20-29 ans |
| 30-39 | 30-39 ans |
| 40-49 | 40-49 ans |
| 50-59 | 50-59 ans |
| 60-69 | 60-69 ans |
| >70 | >70 ans |
| Where are you based? | Dans quelle région du monde vivez-vous ? |
| Asia | Asie |
| Africa | Afrique |
| Central America | Amérique du Nord |
| South America | Amérique du Sud |
| Europe | Europe |
| Eastern Europe | Europe de l’est |
| Middle East | Moyen Orient |
| Oceania | Océanie |
| Your area? | Quel est votre statut ? |
| Medical student | Etudiant dans le domaine de la santé |
| Medical doctor | Professionnel dans le domaine de la santé |
| What is your specialization? (only available to medical doctors) | Quelle est votre profession de santé ? |
| From which university did you (or will you) obtain your highest medical degree? | Dans quelle université (ou école) avez-vous obtenu (ou allez-vous obtenir) votre diplôme le plus élevé ? |
| Where did you (or from where will you) obtain your highest medical degree? | Où l’avez-vous obtenu (ou allez-vous l’obtenir) ? |
| Asia | Asie |
| Africa | Afrique |
| Central America | Amérique du Nord |
| South America | Amérique du Sud |
| Europe | Europe |
| Eastern Europe | Europe de l’est |
| Middle East | Moyen Orient |
| Oceania | Océanie |
| When did you (or will you) obtain your professional medical degree (i.e., highest professional degree)? | Quand avez-vous obtenu (ou allez-vous obtenir) votre diplôme de professionnel de santé le plus élevé ? |
| How familiar are you with… | Dans quelle mesure êtes-vous familier avec... |
| Artificial intelligence | Intelligence Artificielle |
| Machine Learning (ML) | Apprentissage Automatique ou *Machine Learning (ML)* |
| Supervised ML | *ML* Supervisé |
| Unsupervised ML | *ML* non Supervisé |
| Deep Learning | Apprentissage Profond ou *Deep Learning* |
| Natural network(s) | Réseaux naturels ou *Natural networks* |
| Fuzzy logic | Logique floue ou *Fuzzy logic* |
| Support vector machine | Machine à Vecteurs de Support ou *Support Vector Machine* |
| Overfitting/underfitting | Surapprentissage ou Sous-apprentissage |
| Feature selection | Sélection de paramètres ou *Feature selection* |
| Have you attended a course on artificial intelligence ? | Avez-vous déjà suivi à un cours sur l’intelligence artificielle ? |
| Never | Jamais |
| This year | Cette année |
| Last year | L’année dernière |
| Two to three years ago | Il y a deux ou trois ans |
| More than three years ago | Il y a plus de trois ans |
| Would you benefit from more training to: | Pensez-vous nécessaire de bénéficier d'une formation plus approfondie pour : |
| Better understand the main concept of AI | Mieux comprendre le concept principal de l’intelligence artificielle. |
| Explore new opportunities offered by AI in general | Explorer les nouvelles possibilités de l'intelligence artificielle en général. |
| Explore new opportunities offered by AI in medicine and in my field | Explorer les nouvelles opportunités de l'intelligence artificielle dans le domaine de la santé et dans mon domaine. |
| Know more of existing commercial solutions | En savoir plus sur les solutions commerciales existantes. |
| Create my own AI algorithms or applications | Créer mes propres algorithmes ou applications d'intelligence artificielle. |
| In the context of healthcare, who else would benefit from more training and educational programs on AIM? | Dans le domaine de la santé, qui d'autre pourrait bénéficier de formations et de programmes éducatifs supplémentaires sur l’intelligence artificielle en santé ? |
| Medical students | Etudiants en cursus de santé |
| Residents | Internes en médecine, pharmacie ou odontologie |
| Practicing physicians | Professionnels de santé en exercice |
| General public / patients | Le grand public / les patients |
| Hospital administrators | Administrateurs d'hôpitaux |
| Policymakers | Décideurs politiques |
| Caregivers | Le personnel soignants |
| In your field, which of the following issues are important for the development and implementation of AIM? | Dans votre domaine, quelles sont les sujets importants pour le développement et la mise en œuvre de l’intelligence artificielle médicale parmi celles ci-dessous ? |
| Outcomes of AI algorithms are difficult to trace or understand (the blackbox syndrome) | Les résultats des algorithmes d'intelligence artificielle sont difficiles à retracer ou à comprendre (le syndrome de la boîte noire). |
| The complexity of the field of medicine | La complexité du domaine de la santé. |
| The availability of high-quality data samples | La disponibilité d'échantillons de données de haute qualité. |
| The AI's level of autonomy (what AI should and should not do) | Le niveau d'autonomie de l'intelligence artificielle (ce que l'intelligence artificielle doit ou ne doit pas faire). |
| The costs associated with the implementation of AI | Les coûts associés à la mise en œuvre de l'intelligence artificielle. |
| Data privacy / confidentiality | La confidentialité des données. |
| In your field, which factors are the most important for driving the implementation of AIM? | Dans votre domaine, quels sont les facteurs les plus importants pour la mise en œuvre de l'intelligence artificielle médicale ? |
| The availability of comparison studies | La disponibilité d'études comparatives. |
| The safe use of AI | L'utilisation de l'intelligence artificielle en toute sécurité. |
| Build trust between Humans and AI | Instaurer la confiance entre les humains et l'intelligence artificielle. |
| Availability of regulations and legislations | La disponibilité des règlements et des législations. |
| The top management's level of understanding | Le niveau de compréhension de la haute direction. |
| What do you consider the risks of AIM? | Selon vous, quels sont les risques de l'intelligence artificielle en santé ? |
| Dehumanization of healthcare | La déshumanisation des services de soins |
| Reduction in physicians' skills (e.g., physicians might execute fewer types of tasks) | La baisse des compétences des professionnels de santé (par exemple, les praticiens exécuteraient moins de tâches différentes) |
| AI will eventually harm patients | L'intelligence artificielle finira par nuire aux patients. |
| Physicians may become redundant | Les professionnels de santé pourraient devenir superflus. |
| Could you see yourself one day working with an AI algorithm as if it were your colleague? | Vous verriez-vous un jour travailler avec un algorithme d'intelligence artificielle comme s'il s'agissait d’un collègue ? |
| Yes | Oui |
| No | Non |

Table S4 Translation and linguistic validation in French of the questionnaire from Li.

| PERCEPTIONS OF AND BEHAVIORAL INTENTIONS TOWARD LEARNING ARTIFICIAL INTELLIGENCE | |
| --- | --- |
| The original English version | The final version translated into French |
| Personal relevance of medical AI (PR) | Pertinence Personnelle de l'intelligence artificielle médicale (PP) |
| Using medical AI technology enables me to accomplish clinical tasks more quickly | L’utilisation de l’intelligence artificielle me permet d’accomplir mes tâches cliniques plus rapidement |
| Using medical AI technology improves my clinical performance | L’utilisation de l’intelligence artificielle médicale améliore mes performances cliniques |
| Using medical AI technology increases my clinical productivity | L'utilisation de l’intelligence artificielle médicale augmente ma productivité clinique. |
| Using medical AI technology enhances my effectiveness | L'utilisation de l’intelligence artificielle médicale améliore mon efficacité. |
| Subjective Norm related to learning medical AI (SN) | Norme Subjective liée à l'apprentissage de l'intelligence artificielle médicale (NS) |
| My school organizes enrichment lessons for us to learn more about medical AI technologies | Mon école organise des cours d'approfondissement pour nous permettre d'en savoir plus sur les technologies d'intelligence artificielle médicale. |
| My peers and/or parents encourage me to participate in innovative medical AI learning activities. | Mes pairs et/ou mes parents m'encouragent à participer à des activités innovantes d'apprentissage d'intelligence artificielle médicale. |
| My mentors/boss have emphasized the necessity to work creatively using medical AI technology. | Mes encadrants / supérieurs ont insisté sur la nécessité de travailler de manière créative à l’aide d'intelligence artificielle médicale. |
| My classmates feel that it is necessary to learn how to work with medical AI technology. | Mes pairs estiment qu'il est nécessaire d'apprendre à travailler avec l'intelligence artificielle médicale. |
| Perceived Self-Efficacy of learning medical AI (SE) | Efficacité Perçue de l'apprentissage de l’intelligence artificielle médicale (EP) |
| I am certain I can understand the most difficult materials presented in the courses about medical AI. | Je suis certain de pouvoir comprendre les documents les plus difficiles présentés dans les cours sur l'intelligence artificielle médicale. |
| I feel confident that I will do well in clinical practice involving medical AI. | Je suis persuadé que je réussirai dans la pratique clinique impliquant l'intelligence artificielle médicale. |
| I am confident I can learn the basic concepts taught in the courses about medical AI. | Je suis certain de pouvoir apprendre les concepts de base enseignés dans les cours sur l'intelligence artificielle médicale. |
| Basic Knowledge of medical AI (BKn) | Connaissance de Base de l'intelligence artificielle médicale (CB) |
| I understand how computers process medical imaging to produce visual recognition and analysis. | Je comprends comment les ordinateurs traitent l'imagerie médicale pour produire une reconnaissance et une analyse visuelles. |
| I understand how AI technology optimizes the health care solutions | Je comprends comment l’intelligence artificielle optimise les solutions de soins. |
| I understand why AI-assisted genomic diagnostics needs big data for machine learning | Je comprends pourquoi les diagnostics génomiques assistés par l'intelligence artificielle ont besoin de données massives ("big data") pour l’apprentissage automatique |
| I understand how AI assistant in online patient guidance system handle human-computer interaction | Je comprends comment un assistant d’IA, utilisé sur un site ou une application pour orienter les patients, fonctionne dans ses échanges avec l’utilisateur. |
| Behavior alI Intention toward learning medical AI (BI) | Intention Comportementale à l'égard de l'apprentissage de l'intelligence artificielle médicale (IC) |
| I will continue to learn about medical AI technology in the future. | Je continuerai à me renseigner sur la technologie de l'intelligence artificielle médicale. |
| I will pay attention to emerging AI applications used in medical practice | Je prêterai attention aux applications émergentes de l'intelligence artificielle utilisées dans la pratique de la santé |
| I expect that I would be concerned about medical AI development in the future. | Je pense que je prêterai attention au développement de l'intelligence artificielle médicale à l'avenir. |
| I plan to spend time in learning medical AI technology in the future. | J'ai l'intention de consacrer du temps à l'apprentissage de l'intelligence artificielle médicale. |
| Actual Learning of medical AI (AL) | Apprentissage Réel de l'IA médicale (AR) |
| I have intentionally searched and viewed educational videos about medical AI | J'ai intentionnellement cherché et visionné des vidéos pédagogiques concernant l'intelligence artificielle médicale. |
| I have interacted with medical AI applications to understand how they work. | J'ai étudié des applications médicales d'intelligence artificielle pour comprendre leur fonctionnement. |
| I have studied about medical AI through books and journals. | J'ai étudié l'intelligence artificielle médicale dans des livres et des revues. |
| I have attended lessons about medical AI in schools or outside schools. | J'ai assisté à des cours sur l'intelligence artificielle médicale, en présentiel ou en ligne, dans mon établissement ou à l’extérieur. |

Table S5. Translation and linguistic validation in French of the questionnaire from Park.

| PERSPECTIVES ON THE IMPACT OF ARTIFICIAL INTELLIGENCE ON THE PRACTICE OF MEDICINE | |
| --- | --- |
| The original English version | The final version translated into French |
| How big of an impact do you believe that "artificial intelligence" will have on the practice of medicine during your career as a physician? | Quel impact pensez-vous que l'intelligence artificielle aura sur la pratique de la santé au cours de votre carrière ? |
| No significant impact | Pas d’impact significatif |
| Minor - It will impact a few aspects of medicine and surgery | Mineur – Elle impactera quelques aspects de la médecine et de la chirurgie. |
| Moderate - It will impact any aspects of medicine and surgery | Modéré - Elle aura un impact sur certains aspects de la médecine et de la chirurgie. |
| Major - it will impact all aspects of medicine and surgery | Majeur - Elle aura un impact sur tous les aspects de la médecine et de la chirurgie. |
| Which of these specialities do you think will be impacted the earliest and most? | Selon vous, laquelle de ces spécialités sera impactée par l’intelligence artificielle le plus tôt et le plus fortement ? |
| Surgery | La chirurgie |
| Internal medicine | La médecine interne |
| Diagnostic radiology | La radiologie diagnostique |
| Pathology | L’anatomopathologie |
| Dermatology | La dermatologie |
| Family practice | La médecine générale |
| Oncology | L’oncologie |
| Other (please indicate): | Autre : |
| For the speciality that you indicated above, how does the early arrival of "artificial intelligence" in that speciality impact your enthusiasm for going into that speciality? | Pour la spécialité que vous avez indiquée ci-dessus, comment l'arrivée précoce de l'intelligence artificielle dans cette spécialité influe-t-elle sur votre enthousiasme à vous lancer dans cette spécialité ? |
| It makes me much more enthusiastic about going into that speciality | Cela me rend beaucoup plus enthousiaste à l'idée de me lancer dans cette spécialité. |
| it makes me more enthusiastic about going into that speciality | Cela me rend plus enthousiaste à l'idée de me lancer dans cette spécialité. |
| It doesn't impact my enthusiasm for choosing that speciality as a career | Cela n'a pas d'incidence sur mon enthousiasme à choisir cette spécialité comme carrière. |
| it makes me less enthusiastic about going into that speciality | Cela me rend moins enthousiaste à l'idée de me lancer dans cette spécialité. |
| It makes me much less enthusiastic about going into that speciality | Cela me rend beaucoup moins enthousiaste à l'idée de me lancer dans cette spécialité. |
| From which source have you received most of your information about the impact of "artificial intelligence" on your medical career? | D’où tenez-vous la plupart de vos informations concernant l'impact de l'intelligence artificielle sur votre carrière en santé ? |
| My peers (e.g. other medical students or other students in the field) | Mes pairs (par exemple, d'autres étudiants en santé ou d'autres étudiants dans le domaine). |
| My friends (friends who are not in the healthcare field) | Mes amis (amis qui ne travaillent pas dans le domaine de la santé). |
| My mentors/teachers (those who are involved in your education or training) | Mes mentors / enseignants (ceux qui participent à votre éducation ou à votre formation). |
| What I have read from online articles and news stories | Ce que j'ai lu dans les articles en ligne et les informations. |
| What I have read from online forums | Ce que j'ai lu dans les forums en ligne. |
| Movies and TV series | Films et séries télé. |
| Please write a few sentences to describe how what you have heard or read about "artificial intelligence" has impacted your decision or thinking about a sub-speciality. Please include the sub-speciality in your descritpion | Veuillez décrire en quelques lignes comment ce que vous avez entendu ou lu sur l'intelligence artificielle a influencé votre décision ou votre réflexion sur une sous-spécialité. Veuillez inclure la sous-spécialité dans votre description. |
| What year of medical school are you currently in? | En quelle année de formation en école de santé êtes-vous actuellement ? |
| First year | 1ère année |
| Second year | 2ème année |
| Third year | 3ème année |
| Fourth year | 4ème année |
|  | 5ème année |
|  | 6ème année |
|  | Autre : |
